# Hemiarthroplasty vs. open reduction and internal fixation for comminuted distal humerus fractures in patients under 65 years: a systematic review

**DOI:** 10.1016/j.xrrt.2025.07.014

**Published:** 2025-08-09

**Authors:** Cerise Gosselin, Nicolas Bonnevialle, Stéphanie Delclaux, Pierre Mansat, Hugo Barret

**Affiliations:** Chirurgie Orthopédique et Traumatologique, Centre Hospitalier Universitaire de Purpan, Toulouse, France

**Keywords:** Distal humerus fracture, Hemiarthroplasty, Internal fixation, Elbow trauma, Systematic review, Young adult

## Abstract

**Background:**

Comminuted articular fractures of the distal humerus present a significant surgical challenge due to their complexity. Complications are common, and achieving optimal functional outcomes is crucial, particularly in young, active patients. This study aims to compare the outcomes of open reduction and internal fixation (ORIF) with those of hemiarthroplasty (HA) in patients under 65 years old.

**Methods:**

This study is a systematic review and comparative analysis of patients under 65 years of age with comminuted articular fractures of the distal humerus, identified from the existing literature. A comprehensive search of PubMed, Cochrane, and Embase databases was conducted using predefined keywords. Only studies providing detailed individual patient data, including preoperative diagnosis and clinical outcomes after either ORIF or HA, were included. Patients were allocated into 2 groups (ORIF and HA) based on the reported surgical procedure. The primary outcomes assessed were functional results (range of motion, pain (visual analog scale); Disabilities of the Arm, Shoulder, and Hand; and Mayo Elbow Performance scores) and complication rates. The risk of bias and methodological quality of included studies were assessed using the Newcastle-Ottawa Scale.

**Results:**

With an average follow-up of 66 ± 34 months, the 2 groups were comparable in terms of sex, age, follow-up duration, and fracture type according to the Arbeitsgemeinschaft für Osteosynthesefragen classification. The ORIF group included 74 patients (56.8% female, mean age 52 years), and the HA group included 68 patients (70.6% female, mean age 57 years). No significant differences were found between the groups for visual analog scale; Mayo Elbow Performance Score; Disabilities of the Arm, Shoulder, and Hand score; flexion; pronation; and supination. The HA group showed a significantly greater loss of extension (22° vs. 14°, *P* = .013) and a lower flexion-extension arc (97° vs. 109°, *P* = .024). Complications were more frequent in the HA group (54.4% vs. 22.7%, *P* = .002), including stiffness, olecranon hardware discomfort, and clinical instability. The overall revision rate was higher in the HA group (23.5% vs. 8.1%, *P* = .02), mainly due to olecranon hardware revisions (45.4% vs. 0%, *P* < .001). Heterotopic ossifications were also more frequent in the HA group (45.8% vs. 20%, *P* = .027).

**Conclusion:**

For comminuted distal humerus fractures in young patients, ORIF appears to be the preferred surgical option, offering superior functional outcomes and a lower incidence of complications and heterotopic ossification. HA can be used in highly comminuted fractures that cannot be reconstructed with a solid ligament suture or column osteosynthesis and without olecranon osteotomy.

Comminuted articular fractures of the distal humerus (CAFDH) represent a surgical challenge, particularly in young patients under 65 years old. These patients have high functional demands. Such fractures often result from high-energy trauma, such as road accidents or high-impact falls. Surgical intervention is required to achieve the best functional recovery and to attempt to prevent long-term complications such as post-traumatic osteoarthritis of the elbow.

Open reduction and internal fixation (ORIF) is the most frequently performed surgical technique.^1^^,^^11^^,^^13^^,^^15^^,^^20^^,^^32^^,^^37^^,^^45^^,^^49^^,^^53^^,^^70^ It provides satisfactory functional outcomes in approximately 78% of cases.^1^^,^^59^ However, the rate of malunion can reach up to 11% and the rate of nonunion is between 2% and 10%.^25^^,^^44^ Additionally, the rate of stiffness is around 20%,^24^ which is highly disabling in young and active individuals.

Hemiarthroplasty (HA)^2^^,^^3^^,^^27^^,^^48^^,^^57^^,^^61^^,^^65^^,^^66^^,^^71^ represents an alternative for very comminuted and nonreconstructible fractures in young and active patients. A high level of activity and significant functional demands are incompatible with the restrictions associated with total elbow prosthesis.

No study appears to have compared the outcomes of ORIF with HA in young patients. The objective of this study is to compare the clinical and functional outcomes of HA and ORIF in patients under 65 years old with CAFDH. Our hypothesis was that HA could provide comparable results than ORIF in this young population.

## Materials and methods

### Study design

This is a systematic review comparing outcomes of patients under 65 years old with CAFDH. The review was conducted in accordance with the Preferred Reporting Items for Systematic reviews and Meta-Analyses 2020 guidelines.^50^

A systematic search was performed in the PubMed, Embase, and Cochrane databases, covering the period from January 2000 to December 2024, without language restrictions. The search strategy included the MeSH terms and keywords: ("distal humerus fracture"[MeSH] OR "distal humeral fracture") AND ("hemiarthroplasty" OR "open reduction internal fixation" OR "ORIF").

After removal of duplicates, 2 independent reviewers (C.G. and N.B.) screened titles and abstracts, followed by full-text review to determine eligibility. Studies were included if they met all of the following criteria: (1) patients aged 18 to 65 years; (2) comminuted articular distal humerus fracture classified as B3, C2, or C3 according to the Arbeitsgemeinschaft für Osteosynthesefragen (AO)/Orthopaedic Trauma Association classification; (3) treated by either ORIF or HA; (4) minimum follow-up of 12 months; (5) availability of patient-level data enabling extraction of functional outcomes; and (6) study design corresponding to comparative studies, observational studies or case series. Exclusion criteria were: case report, history of ipsilateral fracture or rheumatoid arthritis, open fractures classified as Cauchoix-Duparc^12^ grade ≥2 and/or Gustilo-Anderson^21^^,^^22^ grade ≥ II, and fractures secondary to tumoral lesions. When studies included mixed populations (eg, patients older than 65 years or with fractures other than B3, C2 or C3), only the data corresponding to patients meeting our inclusion criteria were extracted and analyzed.

Data extraction was independently performed by 2 reviewers (C.G., H.B.) using a standardized form that captured demographics (age, sex), fracture type (AO/Orthopaedic Trauma Association), surgical technique, follow-up duration, functional outcomes (visual analog scale (VAS); Disabilities of the Arm, Shoulder, and Hand (DASH); Mayo Elbow Performance Score (MEPS), range of motion), and complications (stiffness, infection, reoperation, ulnar nerve symptoms, hardware issues). Discrepancies were resolved through consensus discussion.

**Figure 1:**
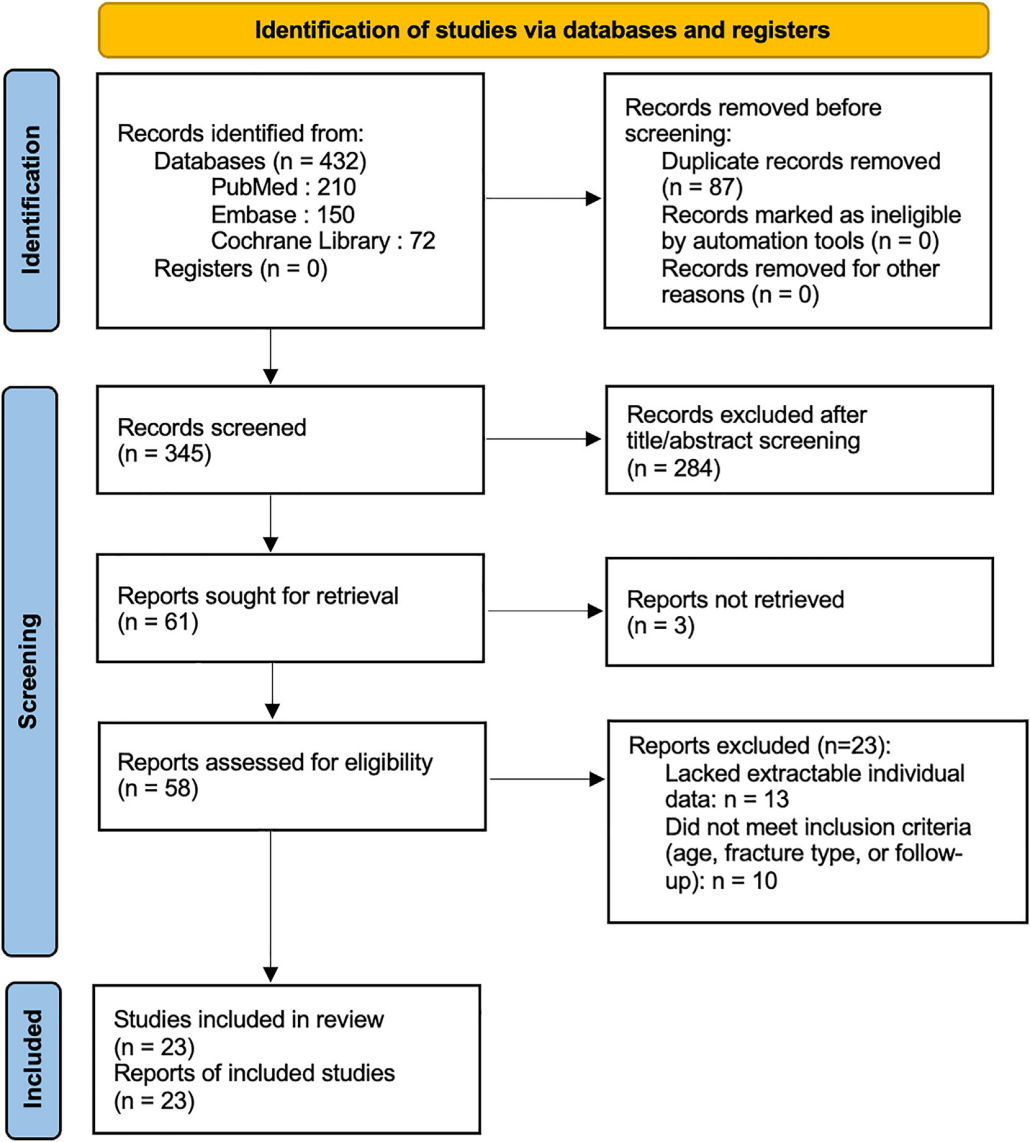
PRISMA flow diagram. *PRISMA*, Preferred Reporting Items for Systematic reviews and Meta-Analyses.

The methodological quality and risk of bias of included studies were independently assessed using the Newcastle-Ottawa Scale. In case of disagreement, a third reviewer (P.M.) was consulted. The study selection process is detailed in the Preferred Reporting Items for Systematic reviews and Meta-Analyses flow diagram (Fig. 1).

### Clinical evaluation

Demographic data were collected, including gender, age at surgery, and follow-up. The type of fracture was specified according to the AO classification.^46^

The clinical assessment included the VAS,^52^ range of motion measurements, and functional scores with the MEPS^6^ and the DASH score.^29^ The range of motion (flexion, extension loss (corresponding to the number of degrees short of full extension (ie, 0°)), flexion-extension (FE) arc; pronation and supination, pronation-supination (PS) arc) was expressed in degrees. A patient was considered to have a functional range if the useful amplitudes according to Morrey et al^43^ were achieved, specifically an FE arc of 100° (30°-130°) and a PS arc of 100° (50° of pronation and 50° of supination).

Complications, as well as reoperations and heterotopic ossifications, were recorded. When available, we documented the surgical approach utilized (with particular attention to whether an olecranon osteotomy was performed) and whether ulnar nerve neurolysis was conducted.

### Statistical analysis

Numerical variables were expressed as mean (± standard deviation) and discrete results as absolute and relative frequencies (%). The comparability of the groups was assessed by comparing the initial demographic data and the duration of follow-up between the groups. The normality and heteroscedasticity of continuous data were evaluated using the Shapiro–Wilk and Levene tests, respectively. Continuous outcomes were compared using ANOVA, Welch's ANOVA, or Kruskal–Wallis tests, depending on data distribution. Discrete outcomes were compared using the chi-square test or Fisher's exact test. The alpha risk was set at 5%, and 2-tailed tests were used. Statistical analysis was performed using EasyMedStat (version 3.33; EasyMedStat, Levallois-Perret, France).

## Results

Detailed data extracted from the included studies are summarized in the supporting documents, organized into 3 separate tables: Table I (ORIF group) and Table II (HA group).

**Table I:** Comprehensive summary of clinical outcomes following treatment of distal humerus fractures by open reduction and internal fixation under 65.

Author (yr)	Study type	N Patients	Mean age (yr)	AO type	Intervention type	Mean follow-up	Main functional scores	Common complications	Surgical revisions
Sur (2020)^70^	Case Series (IV)	17	73.1	Transcondylar	ORIF	≥12 mo	MEPS excellent-good (15/17), flex-ext 117°-20°	Nonunion, ulnar neuropathy	N/A
Hackl (2023)^23^	Case Series (IV)	53	52	B3	ORIF	≥24 mo	MEPS 88, flex-ext 118°	Partial avascular necrosis	2
Proust (2007)^53^	Case Series (IV)	34	77.6	C1, C2, C3	ORIF	35 mo	MEPS 73.3, ROM 80°	Arthrosis (75%), pseudarthrosis (32%)	N/A
Ozer (2005)^49^	Case Series (IV)	11	58.3	C1, C2, C3	ORIF	26 mo	ROM 116° (C1-C2), 85° (C3)	Ulnar neuropathy, ossification	0
Mukohara (2021)^45^	Comparative Study (III)	25	57	Coronal fractures	ORIF	15 mo	MEPS 96.3, flex-ext 10°-130°	Nonunion, avascular necrosis	N/A
Leigey (2014)^37^	Case Series (IV)	15	71	Fragility fractures	ORIF	77 d	Mean ROM 105°	Not specified	0
Kaiser (2011)^32^	Case Series (IV)	16	69	A, B, C	ORIF	30.5 mo	MEPS 84.7, DASH 23.3, flex-ext 16°-129°	Ulnar neuropathy	1
Greiner (2008)^20^	Case Series (IV)	14	55.2	B, C	ORIF	10 mo	MEPS 91, DASH 18.5, flex-ext 121°	Delayed olecranon healing, ulnar neuropathy	0
Celli (2008)^13^	Case Series (IV)	18	Not specified	C2, C3	ORIF	23.1 mo	MEPS good-excellent	Stiffness, residual pain	N/A
Bilsel (2013)^11^	Case Series (IV)	18	45.3	Coronal fractures	ORIF	43.6 mo	MEPS 86.7, DASH 15.3, flex-ext 9°-132.8°	Heterotopic ossification	0
Al-Hamdani (2022)^1^	Case Series (IV)	23	62	C2, C3	ORIF	≥2 y	OES 42, MEPS 85, VAS 2, flex-ext 120°	Pseudarthrosis, stiffness	4

*AO*, Arbeitsgemeinschaft für Osteosynthesefragen; *DASH*, Disabilities of the Arm, Shoulder and Hand Score; *MEPS*, Mayo Elbow Performance Score; *OES*, Oxford Elbow Score; *ORIF*, open reduction and internal fixation; *ROM*, range of motion; *SEV*, Subjective Elbow Value; *VAS*, visual analog scale.

N Patients: Number of patients included in each individual study. AO Fracture Type: Classification of distal humerus fractures according to the Arbeitsgemeinschaft für Osteosynthesefragen/Orthopaedic Trauma Association (AO/OTA). Common Complications: Most frequently reported postoperative complications. Surgical Revisions: Number of revision surgeries or reoperations required within the follow-up period.

**Table II:** Comprehensive summary of clinical outcomes following treatment of distal humerus fractures by hemiarthroplasty under 65.

Author (yr)	Study type	N Patients	Mean age (yr)	AO type	Intervention type	Mean follow-up	Main functional scores	Common complications	Surgical revisions
Al-Hamdani (2019)^2^	Case Series (IV)	24	65	C2, C3	HA	20 mo	MEPS 85, OES 40, flex-ext 110°	Stiffness, residual pain	3
Argintar (2012)^3^	Case Series (IV)	10	66	C3	HA	12 mo	MEPS 81, DASH 30, flex-ext 20°-120°	Pain, stiffness	3
Celli (2022)^14^	Case Series (IV)	41	63	B3, C2, C3	HA	92 mo	MEPS 87.1, DASH 15.9, OES 40.5	Instability, stiffness, ulnar wear	2
Hohman (2014)^27^	Case Series (IV)	8	64	C3	HA	36 mo	MEPS 65-80, DASH 31-39, flex-ext 19°-120°	Hardware pain	4
Nestorson (2015)^48^	Case Series (IV)	42	72	B3, C3	HA	34 mo	MEPS 90, DASH 20, flex-ext 23.5°-126.8°	Stiffness, instability	4
Phadnis (2016)^44^	Case Series (IV)	16	78.7	B3, C2, C3	HA	35 mo	MEPS 89.6, DASH 11.2, OES 43.7, flex-ext 116°	Ulnar wear	0
Rotini (2023)^57^	Case Series (IV)	27	64	B3, C2, C3	HA (Latitude)	>12 mo	MEPS 89.3, DASH 12.6, OES 42.3	Stiffness	0
Schultzel (2022)^53^	Case Series (IV)	10	71.9	C3	HA	115 mo	MEPS 88, DASH 37.1, flex-ext 36°-126°	Fracture, painful hardware	0
Smith (2016)^64^	Case Series Young (IV)	6	44	C3	HA	81 mo	MEPS 88, QuickDASH 12, SEV 89	Aseptic loosening	2
Smith (2013)^65^	Case Series (IV)	26	63	B2.3, B3, C3	HA	80 mo	MEPS 90, QuickDASH 19, flex-ext 116°	Ulnar neuritis, stiffness	4
Stephens (2020)^66^	Case Series (IV)	12	Not specified	N/A	HA	44 mo	MEPS 76.1, PREE 41	Moderate pain	1
Taylor (2021)^71^	Case Series (IV)	7	72.1	C3	HA	29.9 mo	MEPS 88.3, flex-ext 21°-135°	Ulnar neuropathy	1

*AO*, Arbeitsgemeinschaft für Osteosynthesefragen; *DASH*, Disabilities of the Arm, Shoulder and Hand Score; *QuickDASH*, Shortened version of DASH score; *HA*, Hemiarthroplasty; *MEPS*, Mayo Elbow Performance Score; *OES*, Oxford Elbow Score; *SEV*, Subjective Elbow Value; *PREE*, Patient Rated Elbow Evaluation.

N Patients: Number of patients included in each individual study. AO Fracture Type: Classification of distal humerus fractures according to the Arbeitsgemeinschaft für Osteosynthesefragen/Orthopaedic Trauma Association (AO/OTA). Common Complications: Most frequently reported postoperative complications. Surgical Revisions: Number of revision surgeries or reoperations required within the follow-up period.

Percentages reported in Table III and Table IV reflect the proportion among patients with available data for the specific variable, as not all studies reported every outcome for all patients.

**Table III:** Demographic characteristics and clinical outcomes.

Demographic and clinical parameters	ORIF (n = 74)	HA (n = 68)	*P* value
Sex n (%)			.125
Male	32 (43.2%)	20 (29.4%)	
Female	42 (56.8%)	48 (70.6%)	
Age yr, mean (±SD)	52 (21-65)	57 (29-65)	.08
Follow-up mo, mean (±SD)	60 (24-150)	71 (24-151)	.052
AO Classification n (%)			.074
B3	18 (24.3%)	27 (39.7%)	
C2/C3	56 (75.7%)	41 (60.3%)	
Ulnar nerve neurolysis n (%)	51 (68,9%)	68 (100%)	<0,001
Olecranon osteotomy n (%)	32 (43,2%)	23 (33,8%)	<0,001
VAS mean (±SD)	1.1 (±1.6)	1.2 (±1.9)	.894
MEPS mean (±SD)	87.5 (±15.1)	85.7 (±18.1)	.945
DASH mean (±SD)	17.1 (±16.9)	20.3 (±18.5)	.512
ROM degrees, mean (±SD)			
Flexion	125 (±23)	118 (±21)	.06
Extension Loss	14 (±12)	22 (±16)	**.013**
Flexion-Extension Arc	109 (±24)	97 (±28)	**.024**
Pronation	74 (±20)	76 (±18)	.334
Supination	74 (±20)	74 (±21)	.741
Pronation-Supination Arc	150 (±33)	151 (±36)	.497
Functional Range	34 (70.8%)	38 (57.6%)	.21

*VAS*, visual analog scale; *MEPS*, Mayo Elbow Performance Score; *DASH*, Disabilities of the Arm, Shoulder, and Hand; *ROM*, range of motion; *SD*, standard deviation; *ORIF*, open reduction internal fixation; *HA*, hemiarthroplasty; *AO*, Arbeitsgemeinschaft für Osteosynthesefragen.

Sex: Distribution of patients by gender. Age: Age range of patients at the time of surgery. Follow-up: Duration of patient follow-up in months. AO Classification: Classification of fractures according to the Arbeitsgemeinschaft für Osteosynthesefragen system. Functional Range: Number and percentage of patients achieving functional range of motion as defined by specific criteria.

Percentages are calculated based on the number of patients for whom specific data were available, not always on the total cohort.

Bold values indicate statistical significance (*P* < .05).

**Table IV:** Comparison of complications and reoperations between patients treated with ORIF and those treated with HA.

Complications and reoperations	ORIF (n = 74)	HA (n = 68)	*P* value
Complications n (%)	10 (22.7%)	37 (54.4%)	.002
Excluding olecranon osteotomies	10 (23.8%)	35 (51.5%)	.008
Stiffness	9 (15.5%)	22 (32.3%)	.048
Ulnar neuropathy	7 (9.4%)	12 (17.6%)	.217
Olecranon hardware discomfort	9 (22.3%)	10 (45.4%)	.014
Instability	0 (0%)	5 (7.3%)	.023
Humeral nonunion	3 (4.2%)	3 (7.3%)	.666
Reoperations n (%)	6 (8.1%)	16 (23.5%)	.021
Excluding olecranon hardware	6 (8.1%)	11 (18%)	.117
Humerus hardware removal	3 (4%)	0 (0%)	.246
Conversion to TEA	1 (1.3%)	4 (5.9%)	.194
Neurolysis ± transposition of the ulnar nerve	1 (1.3%)	2 (2.9%)	.607
Olecranon hardware removal	9 (22.3%)	10 (45.4%)	.014
Arthrolysis	1 (0%)	3 (4.4%)	.107
Heterotopic Ossifications n (%)	7 (20%)	22 (45.8%)	.027

*ORIF*, open reduction internal fixation; *HA*, hemiarthroplasty; *TEA*, total elbow arthroplasty.

Percentages are calculated based on the number of patients for whom specific data were available, not necessarily on the entire cohort size.

### Patients

The ORIF group included 74 patients with a mean follow-up of 60 months (range 24-150), and the HA group included 68 patients with a mean follow-up of 71 months (range 24-151).

The 2 groups were comparable regarding sex, age, follow-up duration, and fracture type according to the AO classification (Table III). No significant difference was found between the 2 groups concerning fracture type according to the AO classification.

### Surgical technique

In the ORIF group, the most frequently used approaches were olecranon osteotomy (43.2%), V-Y approach (24.3%), and extensile lateral approach (20.3%). Neurolysis of the ulnar nerve was performed in 77.3% of cases (Table III).

In the HA group, the main approaches were olecranon osteotomy (58.9%), triceps-on approach (20.5%), and triceps split approach (15.4%). The ulnar nerve was neurolyzed in all cases.

### Outcomes

The loss of extension, defined as the number of degrees short of full extension (0°), was significantly greater in the HA group (22 ± 16°) than in the ORIF group (14 ± 12°; *P* = .013). The FE arc was significantly greater in the ORIF group compared to the HA group, with 109 ± 24° and 97 ± 28°, respectively, *P* = .024. VAS, flexion, pronation, supination, MEPS, and DASH scores were not significantly different between the groups.

### Complications

Complications were more frequent in the HA group compared to the ORIF group (54.4% and 22.7% respectively, *P* = .002). After excluding olecranon osteotomies, the complication rates remained higher in the HA group (23.8% in the ORIF group and 51.5% in the HA group, *P* = .008) (Table IV).

The rates of stiffness (32.3% vs. 15.5%, *P* = .048), discomfort due to olecranon hardware (45.4% vs. 22.3%, *P* = .014), and prosthetic/elbow instability (7.3% vs. 0%, *P* = .023) were higher in the HA group.

There was no difference in the rate of ulnar nerve neuropathy between the groups.

The overall revision rate was significantly higher in the HA group than in the ORIF group (23.5% and 8.1% respectively, *P* = .02) due to reoperations for hardware removal. Only the rate of olecranon hardware removal showed a significant difference between the 2 groups (45.4% vs. 22.3%, *P* = .014).

### Radiological results

The rate of humeral nonunion was not different between the groups.

The HA group had significantly more heterotopic ossifications compared to the ORIF group, with 45.8% vs. 20% respectively, *P* = .027.

## Discussion

Distal humerus fractures are relatively common, with an incidence of 5.8 to 8.3 fractures per 100,000 per year.^10^^,^^56^ Upper limb surgeons as well as trauma surgeons will therefore treat these injuries in their practice. Essential objectives for achieving good outcomes include anatomical reconstruction of the articular surface and stable fixation allowing for early functional mobilization. ORIF provides satisfactory results in 61% of cases.^35^ However, the rate of malunion can reach 30%, and nonunion occurs in 2% to 10% of cases.^26^^,^^33^ With an average follow-up of 66 ± 34 months, the study found no significant differences in VAS, MEPS, and DASH scores between the 2 groups. Although extension loss and the FE arc were significantly better in the ORIF group (−6° and +12°, respectively), these values are below the MCID of 25°^40^^,^^69^ and therefore not clinically relevant. No significant differences were observed in terms of flexion, pronation, supination, and PS arc between the 2 groups. Comparison of complications revealed significantly higher overall complication rates in the HA group (54.4%) compared to the ORIF group (22.7%). Specifically, the rates of stiffness (32.3% vs. 15.5%), discomfort due to olecranon hardware (45.4% vs. 22.3%), and instability (7.3% vs. 0%) were significantly higher in the HA group. The overall revision rate was significantly higher in the HA group (23.5%) compared to the ORIF group (8.1%), with higher rates of olecranon hardware removal (45.4% vs. 22.3%).

Our results are consistent with the literature. Hackl et al^23^ reported on 53 patients treated with ORIF (mean age 52 years, minimum follow-up 2 years) and found a mean MEPS of 88 points, a mean VAS of 2, and a FE arc of 118°, which is comparable to the functional outcomes in our ORIF cohort. Similarly, Smith et al^64^ analyzed HA in 6 patients under 55 years (mean age 44 years), reporting a mean MEPS of 88, DASH 12, and a FE arc of 101°. These results align with our HA group. However, their complication (50%), stiffness (16.7%), loosening (33.3%) and reoperation rates (66.7%, including 66% olecranon hardware removal and 33.3% conversion to total elbow arthroplasty (TEA) were higher than those observed in our review.

Olecranon osteotomy was a common cause of reoperations in the HA group. For elbow arthroplasties, whether total or partial, it is preferable to avoid approaches that disrupt the extensor tendon.^67^ Current consensus in elbow arthroplasty further endorses the lateral paraolecranon technique.^68^ It allows direct visualization of the articular surface through medial and lateral triceps windows while maintaining soft tissue integrity.^5^ Consequently, postoperative recovery—particularly active extension—is facilitated, and the risk of triceps insufficiency or secondary hardware removal is reduced.^30^ Moreover, in challenging scenarios such as distal humeral HA or TEA conversions, preserving the triceps provides a stable soft tissue envelope and may simplify future procedures.^31^ Luciani et al^40^ also recommended avoiding olecranon osteotomy in both total and partial arthroplasties, favoring the "triceps tongue" approach, which has been shown to provide better range of motion and fewer complications compared to olecranon osteotomy.^75^ The triceps acts as a coaptation factor, allowing for intraoperative assessment of prosthesis stability in conjunction with the medial and lateral ligamentous structures. Since HA is a nonconstrained prosthesis, it is crucial to properly reconstruct the columns and ligamentous planes in C2/C3 fractures to minimize the risk of instability.^40^^,^^51^ Particular attention should be paid to bone quality when considering distal humeral HA, as placing a HA implant on severely osteoporotic bone is not recommended due to the risk of instability associated with this nonconstrained prosthesis. However, since osteoporosis is rare in patients under 65 year old, the cases of instability observed in our series are unlikely to be explained by poor bone quality. Finally, olecranon osteotomy compromises potential intraoperative conversion to TEA and makes it very difficult in future surgeries.^67^ Additionally, it increases the reoperation rate due to scarring issues and may accelerate chondral wear at the proximal ulna.^5^^,^^31^

The rate of heterotopic ossifications was twice as high in the HA group compared to the ORIF group (45.8% vs. 20%, respectively, *P* = .027). Celli et al^14^ reported 46% heterotopic ossifications at 92 months following HA in 41 patients with a mean age of 63 years. Heterotopic ossifications were found in 7% to 40% of cases after elbow fractures, most commonly with a posteromedial localization.^9^^,^^17^^,^^76^ Arthroplasty appeared to be a risk factor for heterotopic ossifications.^39^^,^^55^ In our review, most studies did not specify whether heterotopic ossifications were symptomatic or asymptomatic, which limits the ability to assess their true clinical relevance. In a systematic review, Liu et al^39^ found 10% of heterotopic ossifications in TEA, associated with symptoms in 3% of patients. Other risk factors reported in the literature included longer delays in management, more "aggressive" surgeries, delayed mobilization, among others.^7^^,^^9^^,^^17^^,^^28^ These ossifications led to a significant reduction in the FE and PS arcs.^17^^,^^73^ Prophylaxis was often based on the use of nonsteroidal anti-inflammatory drugs such as indomethacin at a dosage of 75 mg twice daily or 25 mg 3 times daily for 3 to 6 weeks postsurgery.^41^^,^^58^ However, in a recent level 1 randomized controlled trial including 164 patients, Atwan et al^4^ demonstrated no significant difference at 1 year between indomethacin and a placebo regarding the incidence of heterotopic ossifications, MEPS score, and complication rates. Li et al^38^ demonstrated the effectiveness of celecoxib at a dose of 200 mg twice daily for 1 month in significantly inhibiting the formation of heterotopic ossifications when administered immediately after surgery. Treatment for functional impairment involves surgical excision, which significantly improves the range of motion.^36^^,^^54^^,^^74^ Outcomes seemed to be better if this excision is performed early, within ≤12 months.^18^^,^^19^^,^^34^^,^^42^

ORIF appeared to yield poor results in cases of intra-articular comminution.^16^^,^^47^^,^^60^ The use of HA should be considered by surgeons experienced in elbow arthroplasty when stable osteosynthesis with early mobilization was not possible and/or there was significant articular comminution. The bone must be of good quality with the possibility of reconstructing the columns and/or collateral ligaments. If the columns cannot be osteosynthesized, ligament reattachment through the HA implant should be performed. Luciani et al^40^ recommend the use of HA in patients under 65 year old with nonreconstructible comminuted intra-articular distal humerus fractures without pre-existing arthritis and with preserved collateral ligaments or reconstructed columns.

The indication for TEA should be minimized in this population due to the very high rates of complications and early loosening reported in young patients.^8^^,^^62^^,^^63^^,^^72^ TEA implants were constrained prostheses with significant restrictions on lifting heavy loads (less than 5 kg, or even 1 kg if repetitive), which greatly impacts the daily lives of young, active patients.

The better outcomes with ORIF compared to HA in patients aged 65 years or younger can be explained by several factors. ORIF allows for a more precise and stable anatomical reconstruction of the joint, facilitating early mobilization and reducing complications. This technique is less invasive, better preserves anatomical and neurological structures, and is better suited to the functional demands of younger, more active patients. Additionally, ORIF results in fewer heterotopic ossifications and surgical revisions, contributing to overall more favorable outcomes. Finally, the technical proficiency of surgeons in performing ORIF is generally higher, reducing variability in results.

This study had several strengths. It included a large sample size, which enhances the statistical power and reliability of the findings. Additionally, the study benefited from a mid-term follow-up period, averaging 66 months. This was the first study to compare the outcomes of HA and ORIF in patients under 65 year old, addressing a significant gap in the existing literature.

This study has certain inherent limitations typical of retrospective systematic reviews, including potential publication bias, methodological heterogeneity among source studies, and variations in surgical techniques used. However, rigorous analyses of group comparability and critical assessment of the risk of bias were conducted to minimize their impact.

## Conclusion

For comminuted distal humerus fractures in young patients, ORIF is preferable for better functional outcomes, fewer complications, and heterotopic ossifications. HA can be considered in cases of highly comminuted fractures that cannot be reconstructed or stabilized with osteosynthesis, provided that there is a solid ligamentous suture or intact or reconstructed columns, and ideally without the need for an olecranon osteotomy.

## Disclaimers

Funding: No funding was disclosed by the authors.

Conflict of interest: The authors, their immediate families, and any research foundations with which they are affiliated have not received any financial payments or other benefits from any commercial entity related to the subject of this article.
